# An Integrated Approach for Spatio-Temporal Cholera Disease Hotspot Relation Mining for Public Health Management in Punjab, Pakistan

**DOI:** 10.3390/ijerph17113763

**Published:** 2020-05-26

**Authors:** Fatima Khalique, Shoab Ahmed Khan, Wasi Haider Butt, Irum Matloob

**Affiliations:** Department of Computer and Software Engineering, National University of Sciences and Technology, Islamabad 44000, Pakistan; kshoab@yahoo.com (S.A.K.); wasi@ceme.nust.edu.pk (W.H.B.); irum.matloob@ceme.nust.edu.pk (I.M.)

**Keywords:** cholera dynamics, health data analytic, integrated health modeling framework, public health, spatio-temporal analysis

## Abstract

Public health management can generate actionable results when diseases are studied in context with other candidate factors contributing to disease dynamics. In order to fully understand the interdependent relationships of multiple geospatial features involved in disease dynamics, it is important to construct an effective representation model that is able to reveal the relationship patterns and trends. The purpose of this work is to combine disease incidence spatio-temporal data with other features of interest in a mutlivariate spatio-temporal model for investigating characteristic disease and feature patterns over identified hotspots. We present an integrated approach in the form of a disease management model for analyzing spatio-temporal dynamics of disease in connection with other determinants. Our approach aligns spatio-temporal profiles of disease with other driving factors in public health context to identify hotspots and patterns of disease and features of interest in the identified locations. We evaluate our model against cholera disease outbreaks from 2015–2019 in Punjab province of Pakistan. The experimental results showed that the presented model effectively address the complex dynamics of disease incidences in the presence of other features of interest over a geographic area representing populations and sub populations during a given time. The presented methodology provides an effective mechanism for identifying disease hotspots in multiple dimensions and relation between the hotspots for cost-effective and optimal resource allocation as well as a sound reference for further predictive and forecasting analysis.

## 1. Introduction

With big data in healthcare as well as other domains being generously generated, it presents endless possibilities for creating meaningful public health analytical models. Big data in health comes from multiple health-related interdependent or independent sources including Electronic Health Records (EHR) or Electronic Medical Records (EMR), laboratory tests and results, pharmaceuticals, prescriptions, medical imaging, genomics, etc. [1]. The data acquired are examined for subsequent analysis using predictive modeling and analysis [2,3]. The research investigations and their results have tremendously affected the health care sectors including clinical settings, medical services, pharmaceuticals, public health and other related segments [4,5,6].

In order to meaningfully utilize and manipulate big data in health, it is important to channel its flow from the source to systems where it can be managed, analyzed and used for analytical purposes. With an increase in data analysis, storage and dissemination capabilities, a framework used to acquire, integrate, analyze and distribute data at multiple scales can be implemented. Such a framework is able to support top-down analytical approach for example, from the individual to the gene level, as well as the bottom-up approach, for example, data aggregation from individual to population level. In addition, using the breadth of evidence base will allow representing populations from individual cases and a shift from passive to active surveillance systems in real-time as presented in our prior work. The data are obtained from multiple heterogeneous resources in a standardized way ensuring privacy and security while making it accessible to multiple facilities supporting research and evidence-based public health policy and intervention program designing [7]. Furthermore, there are various types and sources on population data that can be effectively analyzed for meaningful patterns for public health intervention programs while staying privacy-compliant [8,9].

Traditionally, public health data acquisition and analysis use aggregated data. However, many infectious diseases, including cholera, have complex driving factors involved. Given the high variability of disease dynamics with respect to environmental and other driving factors, it is important to study individual case incidences as the evidence base for the population [10]. For instance, in the case of disease management, instead of analyzing a generalized view of disease distribution, we need to identify the time and location where incidences create a dense hotspot or a meaningful pattern.

In this paper, we present a mathematical model to represent diseases and associated factors and operations applied to them to study disease patterns. The purpose of our model is to elicit aspects of a disease in a comprehensible, quantitative and generalized manner. This generalized representation will help to investigate the quantitative dynamics and relationships among diseases and their features. In particular, we study the cholera dynamics in the Punjab province of Pakistan using point process representation and apply data mining techniques to identify cholera hotspots and relationships among the identified hotspots based on selected features.

The rest of the paper is organized as follows. Section 2 gives the related work conducted in a similar manner. Section 3 describes the disease management model, data representation and operations performed to obtain the desired results. The section also outlines the data used to validate the model in the declared study region. Section 4 gives the results and discussion of the results when the proposed methodology is applied to the dataset of cholera incidences.

## 2. Related Work

Mathematical models to represent diseases date back to 1909 and are still widely used to represent disease transmission [11,12,13,14]. Mathematical modeling represents elements of disease transmission dynamics and other candidate factors of interest. The mathematical model construction has allowed applications of solutions from other domains such as network theories [15,16,17], artificial intelligence [18,19], machine learning [20,21,22] and game theories [23,24,25] to disease dynamics. Due to a strong relationship between cholera and other environmental factors, cholera transmission models frequently include other features of interest [26,27,28].

Stochastic or point processes have been effectively applied in studies that involve disease analysis in the population or sub-population in a region. These processes use two or three-dimensional space to represent disease incidences and thus, constitute a spatial study of the incidences [29,30,31,32]. The spatial and spatial-temporal models are different from each other in terms of time. Introducing the element of time into spatial analysis constitutes a spatio-temporal analysis. Spatial analysis is performed using two or three-dimensional space represented as (x,y) or (x,y,z) respectively. The time element can be introduced as third dimension *z*, that is, (x,y,t) or as an additional data feature, that is, [(x,y)+f)] where f is the datetime feature [33]. These representations are frequently utilized in multiple analytical modeling methods including spatial filtering, Bayesian method, cluster identification and regression modeling techniques [34,35,36].

Significant research has been conducted in spatio-temporal analysis for multiple domains including sociology, criminology, economics, biology, health and environmental sciences etc. [37,38,39,40,41,42,43,44]. In the context of health data clustering, using the above-mentioned mechanisms can greatly affect the resulting clusters since the process of clustering is sensitive to space and time scales [45]. Different space and temporal data ranges can result in a different set of clusters. Creating spatial hotspots is a relatively straightforward task with many successful statistical techniques available including Getis–Ord Gi* [46], local Moran’s I [47], score statistic [48], and independent component analysis [49] etc. In addition to statistical techniques, data mining attempting to uncover previously unknown interesting patterns in the disease data are also applied. These techniques, although based on statistical foundations, integrate data mining including Machine Learning (ML) and Artificial Intelligence (AI) based methods [50,51,52]. However, when there are other factors affecting the disease incidence, it becomes a complex phenomenon [53]. The candidate driving factors can be incorporated into the model as a third feature or a set of features. For example, factors such as population, socio-economic status, education, water sanitation index, pollution index can be incorporated in the framework one at a time. Combining all candidate features of interest in disease incidence representation for spatio-temoral analysis is not as straightforward and requires a systematic approach towards modeling and analyzing the complex phenomenon.

We introduce a layer-based approach for similar hotspots cluster detection or hotspot relationship mining based on correlation identified factors. We use a multi-step process involving partition-based hotspot detection and distance-based similarity approach for mining relationships between the hotspots. Our approach allows identification of similar hotspots based on multiple determinants or factors that are related to each other in a spatio-temporal region. The model presented contributes towards disease modeling and understanding its spatial, temporal dynamics as well as dynamics of other candidate factors driving or contributing to disease spread and their patterns over time. Specifically, the case study presented describes the mechanism for hotspot detection and their evolution over time given a geographic area. In addition, the work contributes towards assessing the similarity of different hotspots based on selected features of interest. This can lead to actionable information including simulation of hotspots trajectories to evaluate the impact of public health interventions given different spatio-tempoiral scales.

## 3. Methods and Materials

### 3.1. Study Area

**Figure 1:**
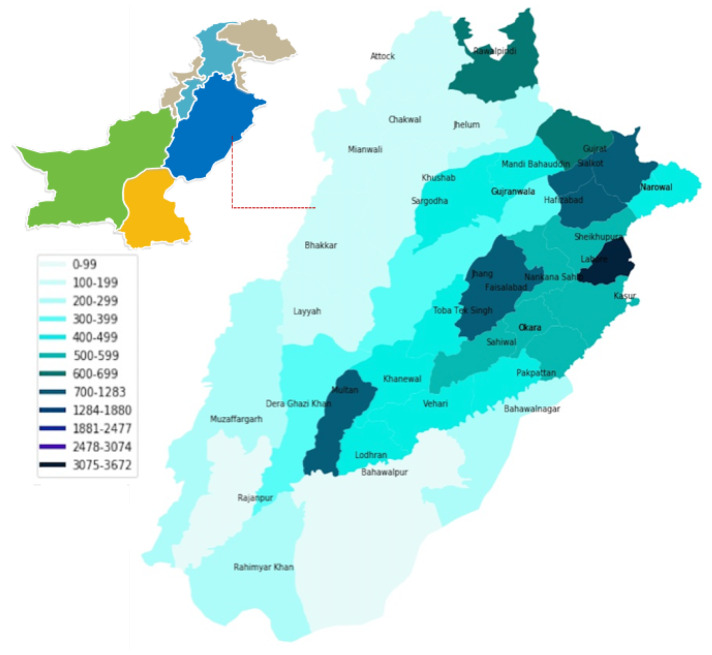
Punjab, a province of Pakistan, is identified as the study area with population density for different districts. Punjab is the highest populus province of Pakistan with 38 districts.

This study was conducted for the cholera outbreak during 2015 to 2019 in the Punjab province of Pakistan. Punjab is the most populated province of Pakistan with a population of over 110 million and a population density of 536 persons per square kilometers shown in Figure 1. The urban population is about 40 million and rural up to 70 million according to Pakistan Bureau of the Statistics Census Results 2017 [54]. The varying dynamics of climatic conditions and an increasing urbanization rate of the province have put a burden on its water resources and has resulted in increased water-related and water-borne diseases including cholera. In addition, Punjab has a complex network of rivers, lakes and water bodies and has a history of flood incidences spawned over years [55].

### 3.2. Dataset

Cholera remains a global threat for public health. It is caused due to contaminated water or food that results in an acute diarrhea and can lead to death within hours if not taken seriously. It has been estimated that up to four million of the world population suffer from cholera annually and up to 143 thousand people die [56]. The data set for cholera incidents in Punjab, Pakistan is obtained from the passive disease surveillance system operated by Punjab Information Technology Board (PITB) [57] where data is reported through all levels of health authorities in Punjab as well as from online portal, mobile application and WHO reports. The data consist of disease name, patient location, date and time for disease incidences from 2015–2019 over multiple locations over all the districts in Punjab province. There were 40,332 cases reported during the time frame. We applied pre-processing steps to the data including, removal of records that had any of the three attributes missing, since they were necessary for spatio-temporal analysis. 35,720 records are obtained based on the pre-processing steps.

The population data were obtained from Federal Bureau of Statistics in Pakistan [54], whereas features of interest including weather attributes are obtained from World Weather Online [58] based on one reading per day, that is, an interval of 24 h using WorldWeatherOnline historical weather data API wrapper v 0.0.4 into pandas v.0.25.1 frame and csv files in python version 5.7.8. The data is downloaded through the API using latitude and longitude of each geocoded disease incidence. Data from the year 2015 to 2019 were analyzed. The presented modeling framework described in Section 3.3 is applied to the case reports from 2015-2019 to underlying incidence cases from the study region of 38 districts.

### 3.3. Disease Management Model

**Figure 2:**
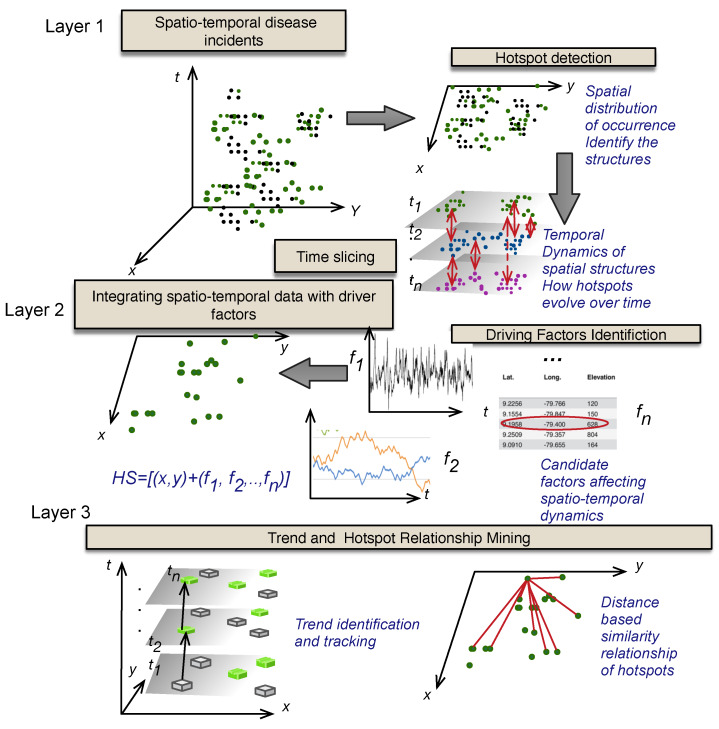
The conceptual model for disease management involving complex interdependent driving factors integrated over spatial and temporal regions. At Layer 1, the data were divided into a series of time slices and each disease incidence is represented in space by (x,y) in corresponding time slice. Layer 2 represents a single time slice, where additional features of interest are added in a given time frame for particular hotspots. Layer 3 presents the trend of hotspots in each hotspot location over all time slices or identification of similar and different hotspots based on all space and time data analysis.

Mathematical models for disease and epidemiology representation help to study disease infection and transmission dynamics. A sub-population with a single disease infection transmitting to a secondary group of individuals can be represented using Poisson distribution [59,60,61]. However, with increasingly changing infectious disease dynamics, it is apparent that many external factors including environmental attributes, climatic conditions, population demographics, industrialization, living standards and conditions, education and migration etc affect the disease dynamics in a population and make up the determinants of diseases. The relationship between disease incidences and other features of interest or determinants is not, however, very straight forward. Active research is being conducted to evaluate the causal and effectual relationship between the two entities. An effective model for disease dynamics must be able to represent and link, in a meaningful way, all possible factors of interest in addition to clinically available disease data. We present a modeling and analytical framework for public health disease management based on spatio-temporal disease incidence integrated with candidate features for evidence-based public health decision support. An overview of the proposed model is provided in Figure 2. We present here the mathematical definitions used in the model. Formally, we define F, as a set of all factor types important in disease transmission dynamics ranged over by *f* and D as a set of all diseases ranged over by *d*. Table 1 lists variables and their descriptions defined in the model. Each incidence of a disease *d* is defined as a tuple

(1) $$d_{ik}=\left(x_{i},y_{i},t_{k}\right)$$

where $d_{i}$ is an instance of disease incidence at a location, defined by a pair of coordinates (x,y), and *t* is the time of the occurrence incident.

#### 3.3.1. Hotspot Detection

Clustering works as a high performance algorithm for hotspot detection in spatio-temporal data [62,63]. We employ a spatial algorithm for finding dense disease incidence areas called hotspots. We use a partition-based algorithm to find the disease spatial hotspots where a distance function is used to find locations with a high occurrence of diseases called hotspots. The time is scaled based on time step values within the clusters to identifying patterns among clusters. A disease incidence data stream is clustered into *n* clusters where each cluster $C_{j}$ with j=1,…,n is defined by a tuple as in Equation (2).

(2) $$C_{j}=\left(id_{j},H_{j},d_{j}^{\prime },m_{j}\right),$$

where id is the cluster identification, *H* is the centroid or hotspot in the cluster, $d^{\prime }$ is the subset of *d* that belongs to cluster *C* and $m_{j}$ is the number of total disease incidences in cluster $C_{j}$. Each hotspot is represented by a latitude and longitude, that is,

(3) H=(x,y),

where *x*, *y* are represented in the form of longitude and latitude representing location of the hotspot.

#### 3.3.2. Correlation Based Factor Selection

To find the potential causal relationship between the disease incidence and external factors, we measure the relationship between the factors f⊂F and disease incidence using correlation. The sequence d(ik) is a time series sequence at location *i* and is a mapping function from a set of dates with a unit interval, for example, one day. In this case, the time step *L* is defined as

(4) $$L^{k}\left(t\right)=d\left(t-k\right)\;or\;L^{-k}\left(t\right)=d\left(t+k\right),$$

where k=1 for a day interval and k=30 for a month interval. This allows finding auto-correlation of *d* given by (5), as well as cross-correlation of *d* with *F* represented as (6) and calculated using (5).

(5) $$r_{k}=\sum_{t=k+1}^{T}\left(d_{t}{-a}_{d}\right)\left(d_{t-k}{-a}_{d}\right)/{\left(d_{t}{-a}_{d}\right)}^{2},$$

where *T* is the time boundary and $a_{d}$ is the mean of the sequence. The factor values are agrregated over time based on the selected *k* and over space based on the defined hotspot locations. Our goal is to select most significant factors that are affecting the disease burden. For this purpose we find the correlation of each factor independently with the disease burden given by (6)

(6) $$\rho_{k}=d⊕f_{i},$$

where i=1,..|F| and ⊕ is used represent correlation function. We then select factors contributing towards disease burden based on threshold Δρ. Therefore, all factors with correlation greater than $\Delta \rho_{k}$ are contributing towards disease incidences as given by (7).

(7) $$f=\sigma_{c}F,$$

where *f* is a set of all those factors that satisfy the condition $c=\rho_{k}<\Delta \rho_{k}$

#### 3.3.3. Intgrating Spatio-Temporal and Determinant Factors

Each hotspot *H*, given by (8), is defined by its location, in terms of (x,y) and values of set of features *f* satisfying a condition given by (7).

(8) $$H=[\left(x,y\right)+\left(f_{1},f_{2},..,f_{n}\right)],$$

where *n* is the total number of correlated external factors. For example, (8) can represent a cholera disease hotspot at a certain location, (long,lat) with values of temperature, population density, pollution index averaged over *L* where $f_{1}=temperature\_value$, $f_{2}=populationdensity\_value$, f3=pollutionindex_value.

#### 3.3.4. Feature Based Hotspot Relation Mining

Hotspot relation mining is based on neighborhood tagging that involves identifying a pattern in the disease data that is based on multiple determinants. Based on $Ld^{-}k\left(t\right)$ in (4), we define a sequence of consecutive time slices $s_{1},s_{2},\ldots ,s_{T}$. Now that each *H* is defined in terms of multi-dimensional factors, in addition to space and time, we explore each cluster for the defined time slice and tag neighbors. Note that the definition of the neighborhood includes, $x,y,t,+f_{1},f_{2},\ldots ,f_{n}$, that is, the similar intensity of disease cases occurring close to each other geographically and temporally and the features of interest of that area for the given time slice are also similar. In this way, we are able to identify if any given location within a cluster is different from the rest of the areas given all the factor values. This allows us to create a distance matrix and heatmaps for hotspots combining multiple factors of interest that can be analyzed for trend. This constitutes a traditional hotspot analysis but only it is based on more factors than the traditional space and time. For example, at any given location and time, we combine the number of cases, population density, humidity, rainfall and temperature for cholera incidents. We find the similarity between two hotspots based on the distance among the selected factors. In order to avoid skewed results because of different units involved, we calculate the z-score $f^{z}$ for all factor readings as (9)

(9) $$f^{z}=w_{f}-A/S,$$

where $w_{f}$ is the value for feature *f*, *A* and *S* are the mean and standard deviation for the factor *f*. Given two spots $H_{i}$ and $H_{j}$, we find the distance between the locations based the corresponding features in a time slice

(10) $$dist\left(H_{i},H_{j}\right)=\sqrt{\sum_{p=1}^{n}{\left(f_{pi}^{z}-f_{pj}^{z}\right)}^{2}}$$

This distance allows us to cluster similar locations based on selected features. (10) creates a distance matrix M between all the hotspots with elements $M_{ij}$ representing $dist(H_{i},H_{j})$. We define a binary distance matrix $M^{b}$ by defining a threshold $\rho_{th}$ over *M* to determine whether $H_{i}$ and $H_{j}$ are in the same cluster based on $\rho_{th}$.

(11) $$M^{b}=\left\{\begin{array}{cc} 0, & \mathrm{if}\;M_{ij}\geq \rho_{th}\;\mathrm{or}\;i=j\\ 1, & \mathrm{otherwise} \end{array}\right.$$

$M^{b}$ is determined for all time slices and added over the sequence to determine the frequency of each feature over the study period. We then apply threshold over the frequency based matrix to identify four categories of similarity among the hotspots. This allows us to define a graph *G* consisting of non empty set of hotspots represented as node as H(G) and a set of edges *E* represented as pair e=uv where an edge *e* connects u,v⊂H(G) if they are similar and fall in the same similarity group. Therefore, we define the graph *G* as a set containing an ordered pair $H_{i}$ and $H_{j}$ and a relation *R*

(12) $$G=(\left(H_{i},H_{j}\right),R),$$

where R={very similar, similar, different, very different}

Therefore, we are able to identify locations that have similar feature patterns when disease intensity was higher. This allows us to find trends of features in similar locations for the predictive analysis. This also enables us to identify if the neighboring hotspots are similar or different from each other when more determinants are taken into account. The model also gives an important reference for optimized resource allocation and predictive analysis for any disease outbreak based on identified features.

## 4. Results and Discussion

**Figure 3:**
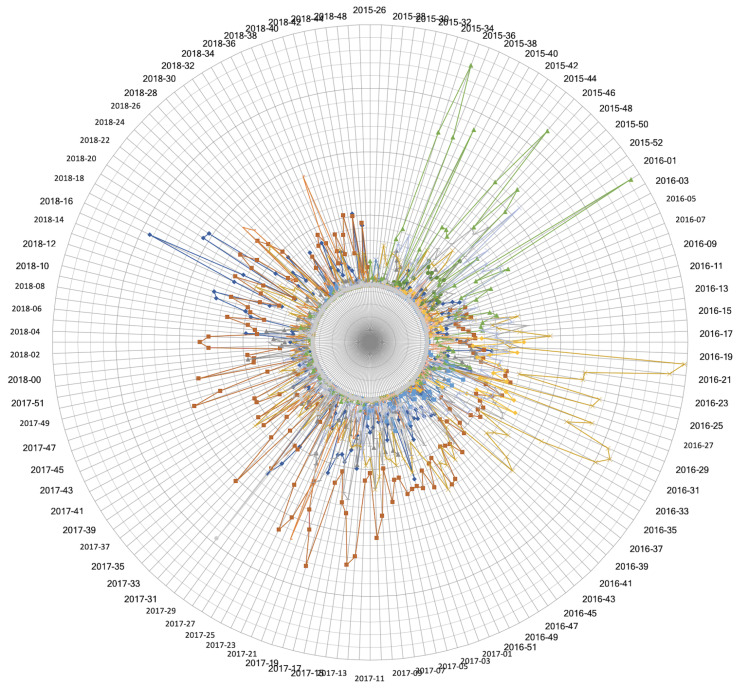
Weekly disease incidence for 15 hotspots.

**Figure 4:**
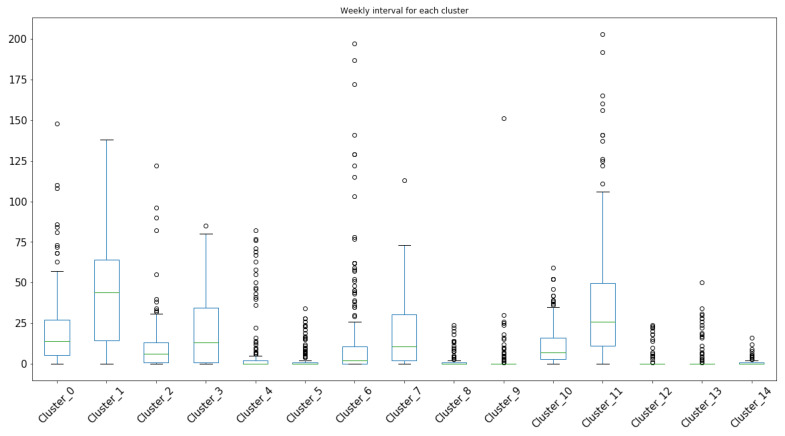
Cholera incidence data distribution in the 15 clusters.

We applied our disease management model to cholera incidence data in 38 districts of Punjab, Pakistan from 2015–2019. We geocode the cholera incident locations to obtain disease incidence dataset using reported incidence location. Each disease incidence is converted to georeferenced point on the map. For identification of high disease incidence hotspots in the province, we used scikit-learn machine learning in Python, a partition-based k-means clustering algorithm and identified 15 locations as cholera hotspots. The weekly distribution of these clusters over time is analyzed. Figure 3 illustrates the incidence rate within each hotspot across the weeks. Figure 4 shows the distribution of disease incidence data in each cluster based on the minimum, first quartile, median, third quartile, and maximum values. We find the cluster centroid location as the hotspot and identify the corresponding tehsil and district based on the coordinates. Table 2 gives each *H* location’s corresponding tehsil and district with a number of cases, that is, *m* in the corresponding cluster. Since the analysis is based on the location of reported incidence data, the administrative area can be identified to any granularity level.

**Figure 5:**
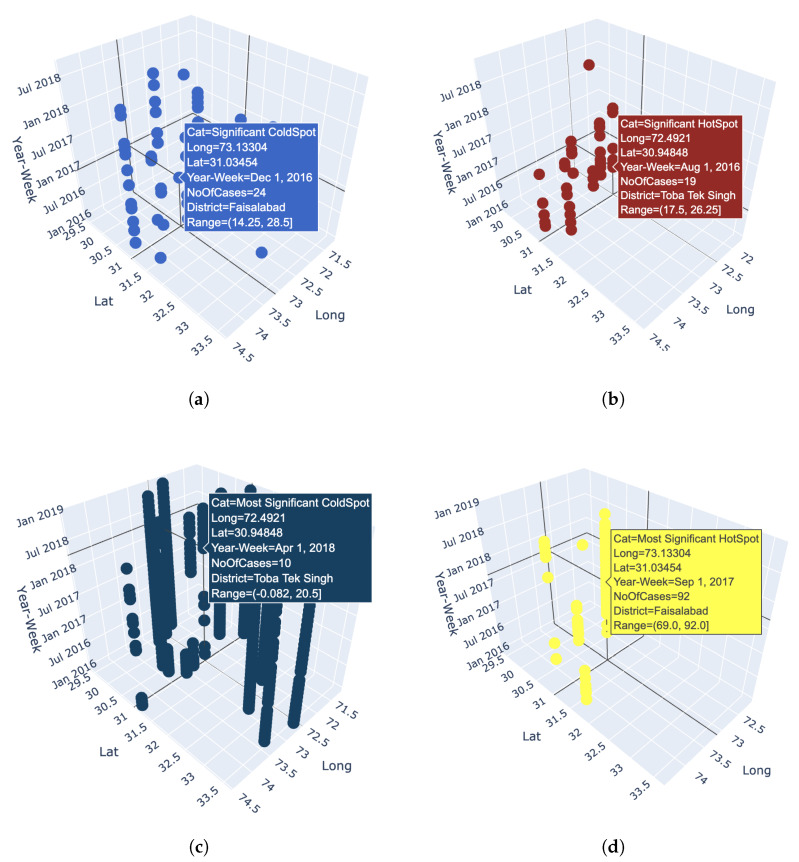
Weekly pattern for four categories of hot and coldspots in at all cluster locations. (**a**) Significant coldspot. (**b**) Significant hotspot. (**c**) Most significant coldspot. (**d**) Most significant hotspot.

**Figure 6:**
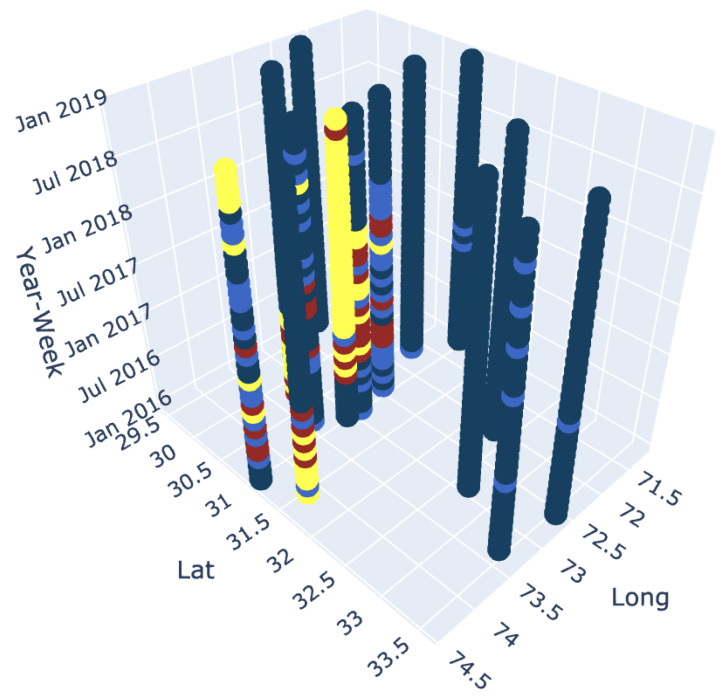
Hotspot trend in an interactive 3D plot over 180 weeks.

In addition, in order to study the trend of each hotspot independently and in relation to other hotspots, each hotspot is analyzed with respect to other hotspots for every week. This allows us to identify the most significant hotspot, significant hotspot, significant cold spot and most significant coldspot every weak among the different hotspots locations. The cholera dataset ranged over 180 weeks. For each week, we classify each hotspot as belonging to the given categories. For example, Figure 5a shows a significant coldspot at week starting from 1 December 2016 with 24 number of cases, at an identified lat long falling under Faisalabad district. Similarly, Figure 5b,c show a significant hotspot in the first week of August in 2016 with 19 number of cases and a most significant cold spot in the first week of April in 2018 with 10 number of cases respectively at Toba Tek Singh district while Figure 5d shows a most significant hotspot at Faisalabad district with 92 number of cases starting in the first week of September in 2017. This creates a pattern of all hotspots over time with a time step equal to a week and defines temporal hotspots for a spatial region as shown in Figure 6. Since the interpretations are more subjective and may not be objectively true, therefore, in order to make maps more meaningful and comprehensible for the decision making personnel, our model must represent the data so that it is objectively true and unbiased to the underlying methodology. The results of the analysis of the weekly hotspots pattern over time in the presented model show that each weak has a different threshold for the number of cases for a hotspot to be assigned to a certain category. Similarly, each hotspot over a period of time, belongs to different categories based on different incidence cases. For example, the hotspot in the Faisalabad district had 92 cases in the first week of September, was a very significant hotspot while the same location also became the most significant hotspot with only 34 cases in the first week of October in 2016. This information is valuable in investigating a location’s trend over time for resource allocation and location dynamics for disease patterns. Furthermore, the analysis can be applied to any spatial and temporal resolutions.

**Figure 7:**
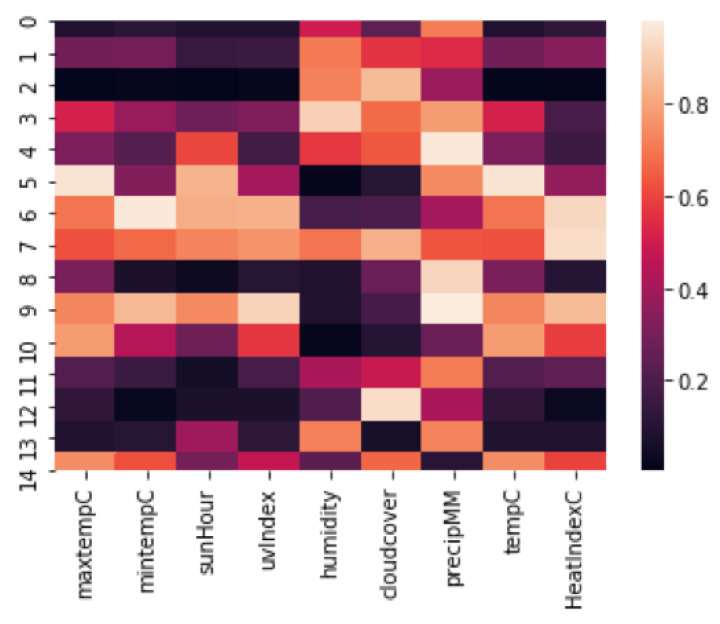
Weekly correlation confidence for nine weather attributes with cholera disease incidence.

**Figure 8:**
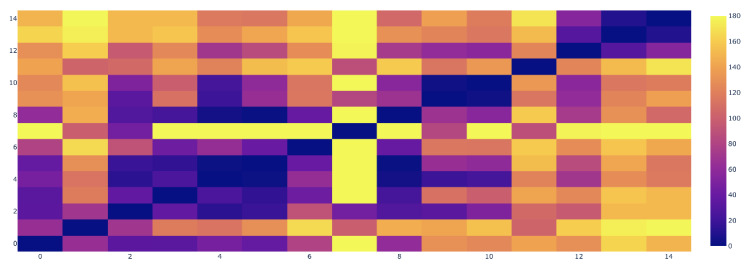
Heatmap for feature-based distance among all hotspot locations.

**Figure 9:**
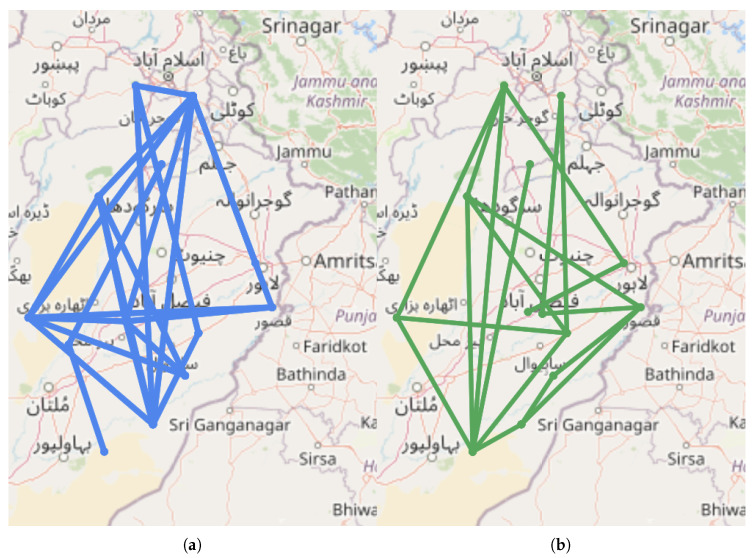

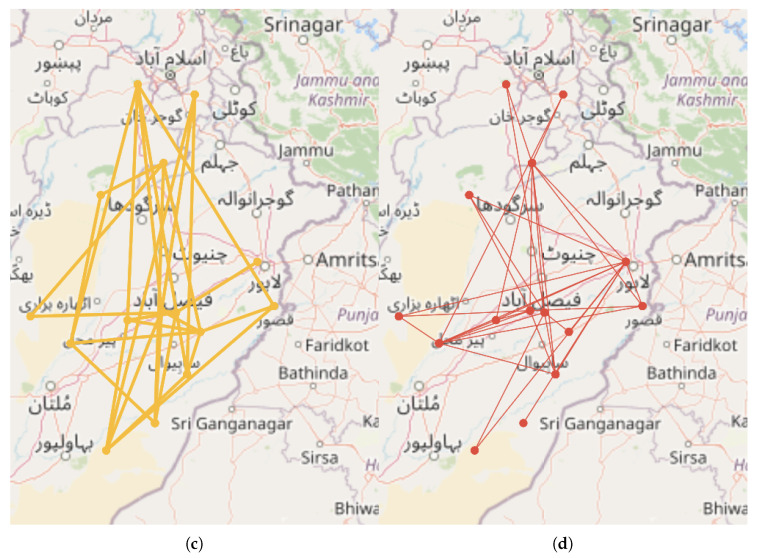
Hotspot relation mining based on features and incidence rate. Width of the line shows similar to different areas. Graph shows location pair connected for: (**a**) Very similar hotspots. (**b**) Similar hotspots. (**c**) Weak similar hotspots. (**d**) Different hotspots.

As additional factors of interest in studying cholera disease patterns, we used nine weather attributes including maximum temperature, minimum temperature, temperature, sun hour, ultraviolet (UV) index, humidity, cloud cover, precipitation and heat index. However, any other related features such as distance of hotspot from water bodies, flood-affected areas and sanitation index can also be included in the model to identify similar hotspots. We calculated z-scores corresponding to the probability of distribution across weeks of data among all clusters. We found a symmetrical set of disese incidences. The points in the normal quantile plot lie reasonably close to a straight line. We used the Pearson correlation coefficient since the data followed the normal distribution. We find the weekly correlation of each weather attribute with the disease incidence at each hotspot. The features with correlation confidence of 95% and above are selected for inclusion in further analysis. Precipitation, humidity and cloud cover were selected as three climatic features having a *p*-value less than 0.05 and correlation confidence greater than 95% (see Figure 7). We also include population density as an external feature for further analysis. We then integrate the determinant factors with disease incidence rate at each hotspot location and find the distance among these locations based on Equations (10) and (11), followed by the summation of the correlation matrices over the weekly range. Figure 8 gives the heat map for distances among the hotspots based on all the candidate factors. Based on these distances, we categorize each pair of locations as different, similar, very similar, and weak similar. Figure 9 shows the final connected pair of hotspots for each category. For example, Table 3 gives a list of 23 location pairs that are very similar to each other in terms of selected features. The tehsil level of these locations was identified, and it comes under the district level in the province. Similarly, 34 location pairs were identified as different, 10 location pairs as similar and 29 location pairs as weak similar based on hotspot relationship mining algorithm.

The analysis of results represented by Figure 7 and Figure 9 show an interesting perspective of the hotspots. There are cities where weather may have played a key role in the spread of Cholera, however, there are others where we find weak or very weak correlation among the disease hot spots with similar weather conditions. This indicates other factors besides weather that must have contributed towards its spread. We have found that many of these hotspots are at locations that are flood-stricken over the selected time-period. The analysis of Figure 9. gives us a reason to look for other factors that may also be included along with traditionally studied factors like floods, construction of major projects, the spread of other diseases, healthcare facilities etc. While other potential factors were not included in this study due to the unavailability of data, the model can take multiple parameters and see their strong and weak-correlation to infer similarities to address the spread. In our case, we have currently taken weather information, but we can easily extend this by considering other factors like floods in the area, socioeconomic conditions and the spread of other diseases. The locations and their corresponding features can be further explored for optimized resource allocation or as a foundation for predictive analysis for forecasting disease patterns in other locations with similar feature patterns.

It is important to allow multiple representations of the analysis to aid the decision-making process. Hotspot analysis presented in this research minimizes the subjectivity present in density-based methods. In addition, the hotspots are statistically significant with respect to the higher number of disease incidences that are based on the z-value. The patterns are identified based on the quantification of confidence. We use z-score for meaningful pattern identification confidence. The quantifiable pattern mining allows important decision-making based on the analysis, such as resource allocation to high disease clusters having a positive impact. When more factors are thrown into the model, the spatial representation does not remain as straightforward as a traditional spatial analysis. The neighboring hotspots in this case may not be in the same spatial neighborhood. The presented model allows a seamless introduction of any number of related features of interest selected through correlation and allows their representation in the model for their inclusion in further analysis.

The presented model framework, however, has its own set of challenges and limitations. First, the identification of hotspots is dependent upon the availability of accurate spatial and temporal data. The data collected in this study were obtained based on hospitals and health care entities under the direct administration of the provincial government. There are many incidences in the region that go unreported, thus affecting the accuracy of the disease distribution model. Second, the incidence addresses and date–time entry method is not standard across all healthcare entities. The obtained data collected needs to be preprocessed for typing or human errors, duplicates and missing information. More than 98% of the dataset needed minor preprocessing since it was obtained through hospital EHR systems and was geocoded successfully to the tehsil and district level. The patient privacy was protected by using minimum tehsil level resolution when identifying hotspots. However, the hotspots identification process used each incidence’s unabstracted location for precise and accurate results.

Infectious public health diseases, including cholera have complex dynamics involving multiple climatic, ecological, social and environmental factors. Our presented work serves as a reference for further investigations involving other determinants of public health interests in the region. For example, with extensive rivers network in the Punjab province, there are multiple flood-stricken areas during the selected time period. Flood data, if supplemented in the model can significantly affect the disease characteristic and pattern mining process results. The availability of relevant data on the required scale can be used by the presented model to accurately provide information for predictive analysis on real-time data to characterize the dynamics of disease in the region.

## 5. Conclusions

The integration of multiple data sources combined with big data technologies enables the exploration of new data relationships. Healthcare data, including disease incidence data combined with data from other sources such as epidemiology data, geographic data, census data, climate data and social determinants data etc., allows conversion of big health data into smart data using data mining and other artificial intelligence techniques. We present a model combining disease incidence data with features of interest in the public-health context for enriching evidence for smart decision support systems. The data elements and operations performed on them reveal disease hotspots, disease trends and hotspot locations categorized based on similarity on the basis of selected features. In addition, one of the major goals of public health policies is to manage cost by optimally utilizing available resources to high disease density areas. The model allows exploration of identified disease hotspots in multiple dimensions that can be continuously enriched by introducing new factors in the model.

## Figures and Tables

**Table 1:** Variables and their descriptions defined in the mathematical model for disease management.

Variable	Description	Variable	Description	Variable	Description
*d*	disease incidence	*m*	cluster size	$f^{z}$	z-score of feature
*C*	cluster	$d^{\prime }$	subset of *d* that belongs to *C*	*M*	distance matrix
*H*	hotspot	*F*	set of all features	$M^{b}$	binary distance matrix
*f*	set of features selected based on correlation	*L*	time step	*R*	relationship between hostspots
*k*	interval length	*A*	mean of sequence	*G*	graph connecting hotspots
ρ	correlation coeffecient	*S*	Standard Varitaion	⊕	cross correlation function
*r*	autocorrelation	σ	conditional operator	$w_{f}$	value for feature *f*

**Table 2:** The 15 hotspots identified with their corresponding tehsil and district as well as the number of cases within each hotspot cluster.

District	Tehsil	No. of Cases
Bahawalnagar	Chishtian	220
Bahawalpur	Khairpur Tamewali	3266
Faisalabad	Tandlianwala	7257
Faisalabad	Sammundri	2699
Jhelum	Choa Saidan Shah	406
Kasur	Kasur	3462
Khanewal	Ahmadpur Sial	321
Khushab	Khushab	3664
Lahore	Ferozewala	1756
Layyah	Layyah	416
Okara	Okara	481
Pakpattan	Pakpattan	123
Rawalpindi	Kallar Sayyedan	1936
Rawalpindi	Fateh Jang	1091
Toba Tek Singh	Toba Tek Singh	6306

**Table 3:** Location pairs with very similar conditions identified scaled to the tehsil level.

S. No	Location A	Location B
1	Chishtian	Layyah
2	Khairpur Tamewali	Ahmadpur Sial
3	Ahmadpur Sial	Choa Saidan Shah
4	Chishtian	Kallar Sayyedan
5	Toba Tek Singh	Khushab
6	Toba Tek Singh	Kallar Sayyedan
7	Toba Tek Singh	Layyah
8	Toba Tek Singh	Kasur
9	Kasur	Kallar Sayyedan
10	Chishtian	Toba Tek Singh
11	Chishtian	Okara
12	Layyah	Khushab
13	Chishtian	Khushab
14	Khushab	Kallar Sayyedan
15	Layyah	Kallar Sayyedan
16	Pakpattan	Khushab
17	Layyah	Kasur
18	Ferozewala	Kallar Sayyedan
19	Pakpattan	Layyah
20	Chishtian	Fateh Jang
21	Pakpattan	Toba Tek Singh
22	Kallar Sayyedan	Fateh Jang
23	Okara	Fateh Jang
